# Identification and genomic analysis of a thermophilic bacterial strain that reduces ammonia loss from composting

**DOI:** 10.1128/spectrum.00763-24

**Published:** 2024-08-20

**Authors:** Xuejuan Chen, Rong Feng, Qianhui Du, Tim H. Mauchline, Ian M. Clark, Yingang Lu, Li Liu

**Affiliations:** 1 College of Agriculture, Guizhou University, Guiyang, Guizhou, China; 2 Lijiang Culture and Tourism College, Lijiang, Yunnan, China; 3 Sustainable Soils and Crops, Rothamsted Research, Hertfordshire, United Kingdom; Institute of Microbiology, Beijing, China

**Keywords:** strain screening, ammonia nitrogen conversion, whole-genome analysis, functional analysis, nitrogen metabolism genes

## Abstract

Ammonia loss is the most severe during the high-temperature stage (>50°C) of aerobic composting. Regulating ammonia volatilization during this period via thermophilic microbes can significantly improve the nitrogen content of compost and reduce air pollution due to ammonia loss. In this study, an ammonia-assimilating bacterial strain named LL-8 was screened out as having the strongest ammonia nitrogen conversion rate (32.7%) at high temperatures (50°C); it is able to significantly reduce 42.9% ammonia volatile loss in chicken manure composting when applied at a high-temperature stage. Phylogenetic analysis revealed that LL-8 was highly similar (>98%) with *Priestia aryabhattai* B8W22^T^ and identified as *Priestia aryabhatta*. Genomic analyses indicated that the complete genome of LL-8 comprised 5,060,316 base pairs with a GC content of 32.7% and encoded 5,346 genes. Genes, such as *gudB*, *rocG*, *glnA*, *gltA*, and *gltB*, that enable bacteria to assimilate ammonium nitrogen were annotated in the LL-8 genome based on the comparison to the Kyoto Encyclopedia of Genes and Genomes (KEGG) database. The results implied that the application of thermophilic ammonia-assimilating strain *P. aryabhatta* LL-8 would be a promising solution to reduce ammonia loss and mitigate air pollution of aerobic composting.

**IMPORTANCE:**

Aerobic composting is one of the essential ways to recycle organic waste, but its ammonia volatilization is severe and results in significant nitrogen loss, especially during the high-temperature period, which is also harmful to the environment. The application of thermophilic bacteria that can use ammonia as a nitrogen source at high temperatures is helpful to reduce the ammonia volatilization loss of composting. In this study, we screened and identified a bacteria strain called LL-8 with high temperature (50°C) resistance and strong ammonia-assimilating ability. It also revealed significant effects on decreasing ammonia volatile loss in composting. The whole-genome analysis revealed that LL-8 could utilize ammonium nitrogen by assimilation to decrease ammonia volatilization. Our work provides a theoretical basis for the application of this functional bacteria in aerobic composting to control nitrogen loss from ammonia volatilization.

## INTRODUCTION

During the heating (to 50°C) and high-temperature (>50°C) stage of aerobic composting, organic nitrogen in organic materials, such as livestock manure and plant straw waste, is easily degraded and converted into ammonium (NH_4_^+^-N) that then accumulates in compost piles (1). Subsequently, most of NH_4_^+^-N is converted to ammonia (NH_3_) and volatilizes as a gas (2, 3). Under high temperature and high pH, NH_3_ volatilization accounted for 44%–79% of total nitrogen loss, which is the main cause of nitrogen loss during composting (4). Various nitrogen retention measures have been developed to effectively reduce the volatilization of NH_3_, including physical (5), chemical (6, 7), and biological (8, 9) methods.

Microbial nitrogen retention additives (MNRAs) can reduce NH_3_ volatilization loss by promoting NH_4_^+^-N conversion through microbial substrate use or nitrogen-converting enzymes’ regulation genes (10, 11). MNRAs are one of the more popular measures to retain nitrogen during composting due to its low cost, easy application, high efficiency, and a lack of pollution. For instance, the application of ammonia-oxidizing bacteria was shown to promote the fixation of NH_4_^+^-N and formation of humus in chicken and straw compost, which was helpful to reduce NH_3_ emission and nitrogen loss (12, 13). The inoculation of ammonia-converting bacteria at the initial stage of pig excrement and wheat straw compost also accelerated organic matter degradation and reduced nitrogen loss (14). The asymbiotic free-living N2-fixing microorganism *Micromonospora sp*. KSC08 could improve microbial activity and regulate nitrogen content in compost mixtures (15). It can be seen that the addition of MNRAs can effectively reduce the nitrogen loss in composting and improve the quality of composting. However, the high temperature of aerobic composting will kill most microorganisms, thereby inhibiting MNRAs. Jiang’s research (14) revealed that no significant effect was observed when the inoculation of ammonia-converting bacteria occurred at the high-temperature stage of composting. Thermophilic ammonia-converting bacteria, which could convert NH_4_^+^-N/NH_3_ to stable N forms, such as nitrate and microorganism nitrogen under high temperature, is a promising MNRA for reducing NH_3_ loss and retaining more nitrogen in composting, which was proved in sewage sludge composting (16) and rural kitchen waste composting (17). Therefore, screening and identifying thermophilic ammonia-converting microorganisms and analyzing their functions have high theoretical and practical significance.

Whole genome sequence analysis of ammonia-converting bacteria is of great guiding significance to understand its functional mechanism in regulating the nitrogen cycle fundamentally. For example, the denitrification function of strain HNDS-6 was confirmed after key nitrification enzymes, including hydroxylamine reductase, nitrite reductase, and nitrate reductase, were identified in its genome sequencing analysis (18). Yan et al. (19) also identified new nitrogen-fixing genes from the genomic analysis of *Pseudomonas stutzeri* A1501. By analyzing the whole genome sequence of *Pseudomonas* G16, Gao et al. (20) found that G16 had enzymes related to the NH_4_^+^-N assimilation pathway, which could assimilate NH_4_^+^-N to synthesize glutamic acid through a series of biochemical reactions, and this provides theoretical support for the application of strain G16 in NH_4_^+^-N transformation.

In this study, a thermophilic bacterial strain LL-8 with high efficiency for NH_4_^+^-N conversion was screened, identified, and sequenced. Genome functions were annotated against the Gene Ontology (GO), Kyoto Encyclopedia of Genes and Genomes (KEGG), Clusters of Orthologous Groups of proteins (COG), and NCBI-nr databases to assess the presence of functional genes related to nitrogen conversion. This study provided a bioinformatic framework for the NH_4_^+^-N transformation mechanism of strain LL-8 and its development and utilization, such as in decreasing ammonia volatile loss in composting.

## RESULTS

### Screening of thermophilic ammonium-converting bacteria with high efficiency

**Fig 1:**
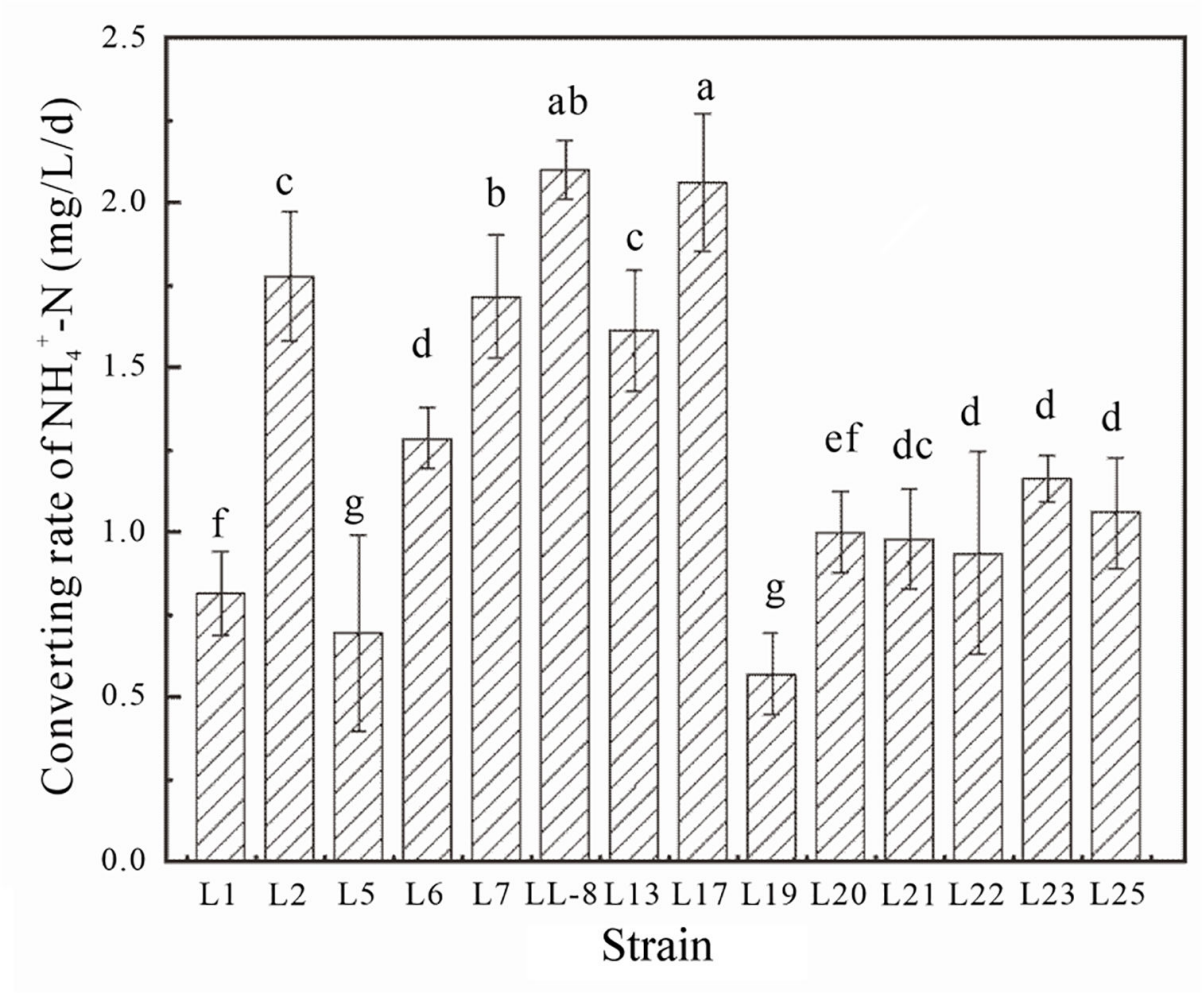
Differences in ammonium nitrogen-converting rates between strains isolated from an aerobic composting matrix during the high-temperature (>50°C) stage.

After 15 days of cultivation, significant differences in ammonium nitrogen (NH_4_^+^-N) conversion rates were observed among the 14 identified strains (Fig. 1). In particular, strains LL-8 and L17 exhibited significantly higher NH_4_^+^-N conversion rates than other strains, whereas strains L5 and L19 exhibited the lowest rates.

**Fig 2:**
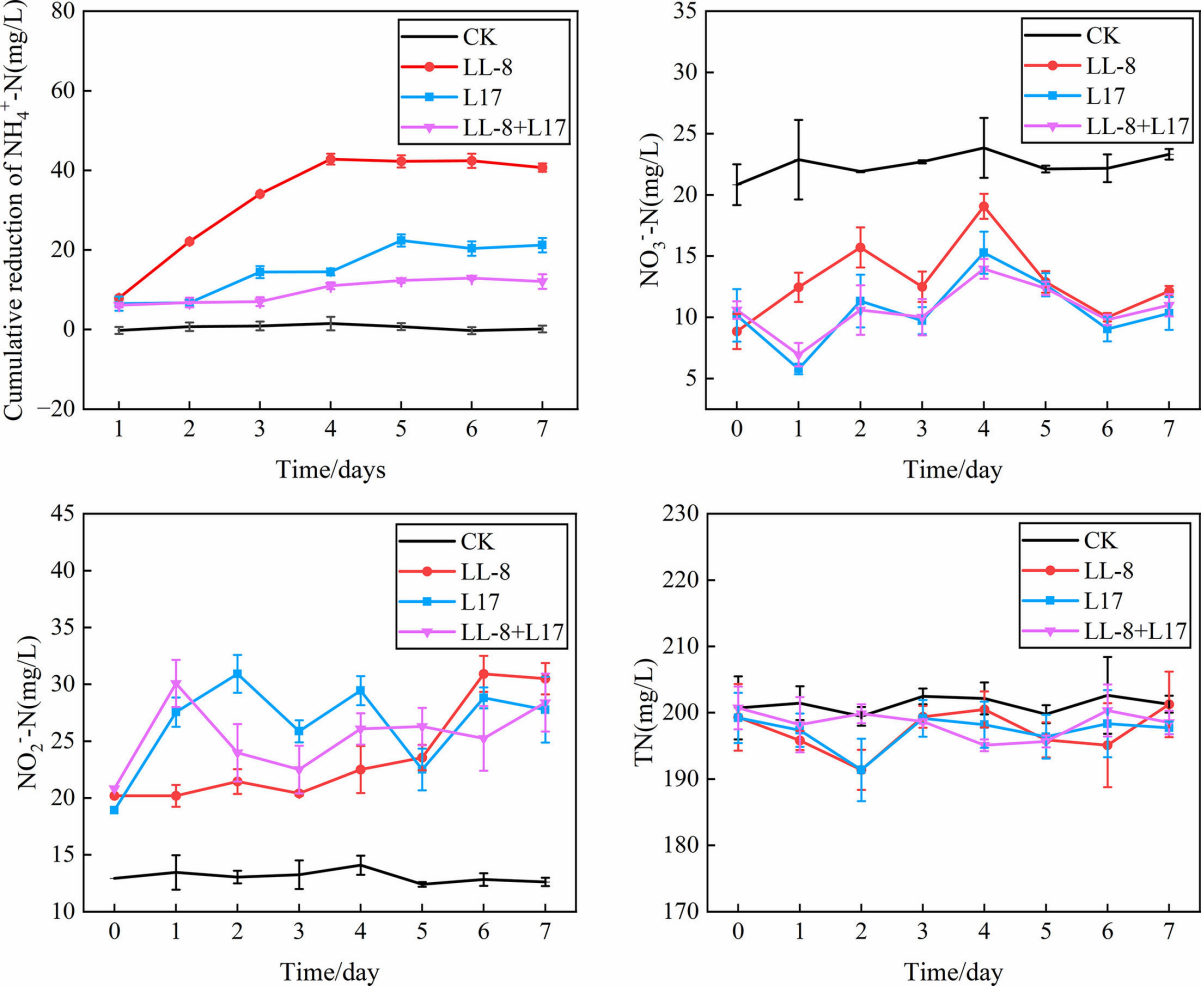
Dynamic changes of NH_4_^+^-N, NO3—N, NO2—N, and total nitrogen (TN) in different bacteria inoculating medium. CK indicates the control treatment without strains, LL-8 indicates the treatment inoculated with LL-8; L17 indicates the treatment inoculated with L17. LL-8+L17 indicates the treatment inoculated with LL-8 and L17 for co-culturing.

The cumulative reduction of NH_4_^+^-N in the culture medium of the strain L17, LL-8, and LL-8+L17 treatments increased with cultivation time and then plateaued (Fig. 2). The maximum cumulative reduction of NH^+^-N appeared after the 4th day of cultivation for strain LL-8, which was double the highest accumulation for L17 on the 5th day. The NH_4_^+^-N conversion efficiency of strain LL-8 was 32.7%, whereas that of L17 was 16.97%. Co-culture of strains LL-8 and L17 led to inhibited conversion of NH_4_^+^-N, with the lowest efficiency of 10.65% observed. The nitrate nitrogen (NO3—N) content of strain LL-8 culture was higher than that of the other two inoculated treatments but lower than that of CK. The nitrite nitrogen (NO2—N) content of the strain LL-8 culture was significantly lower than that of L17 and LL-8+L17 cultures in the first 5 days but higher than that of CK. However, no significant changes in total nitrogen contents were observed among the treatments. Strain LL-8 clearly exhibited higher efficiency in converting NH_4_^+^-N (*P* = 0.0367) and retaining more NO3—N compared with L17 (*P* = 0.002), thus being the most promising candidate to promote NH_4_^+^-N conversion.

### Ammonia volatilization of composting with strain LL-8

**Fig 3:**
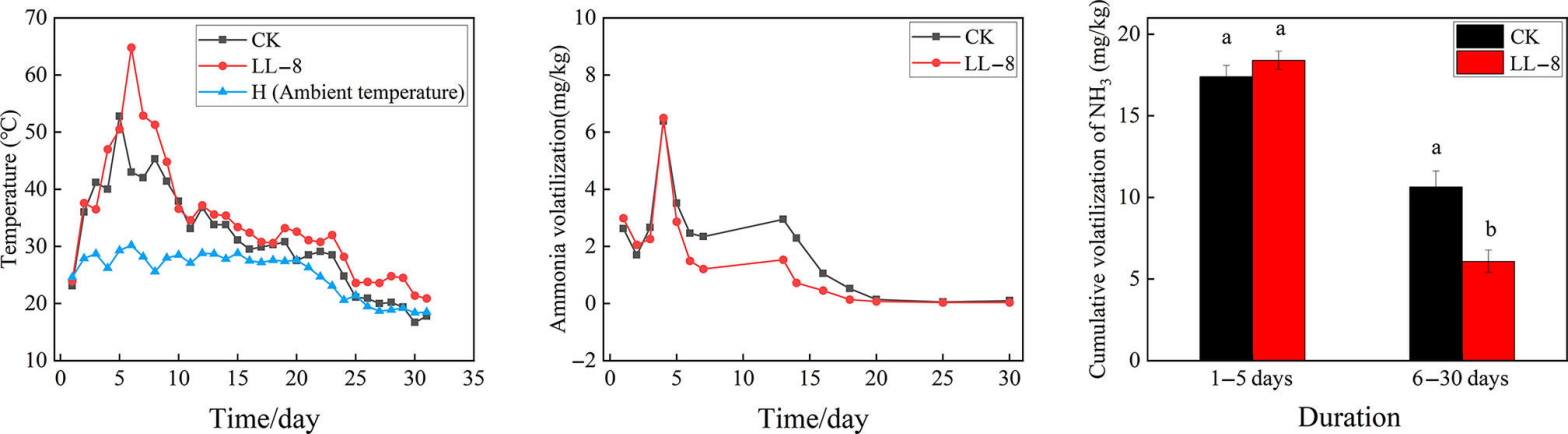
Effect of LL-8 on ammonia volatilization of chicken manure composting when inoculated at the high-temperature stage. CK indicates the control treatment without strains; LL-8 indicates the treatment inoculated with LL-8. H indicates the ambient temperatures.

As shown in Fig. 3, when the temperature in the chicken manure composting increased to 50°C, the NH_3_ volatilization of the treatment inoculated with LL-8 at the high-temperature initial stage was significantly lower than that of CK with sterilized liquid medium. From the high-temperature stage to the end of composting, the cumulative NH_3_ volatilization of LL-8 treatment was 42.9% lower than that of CK (*P* = 0.0284). It is shown that the addition of LL-8 inoculants during the high temperature period of aerobic compost was able to reduce NH_3_ volatilization loss effectively.

### Morphological and phylogenetic analysis of strain LL-8

**Fig 4:**
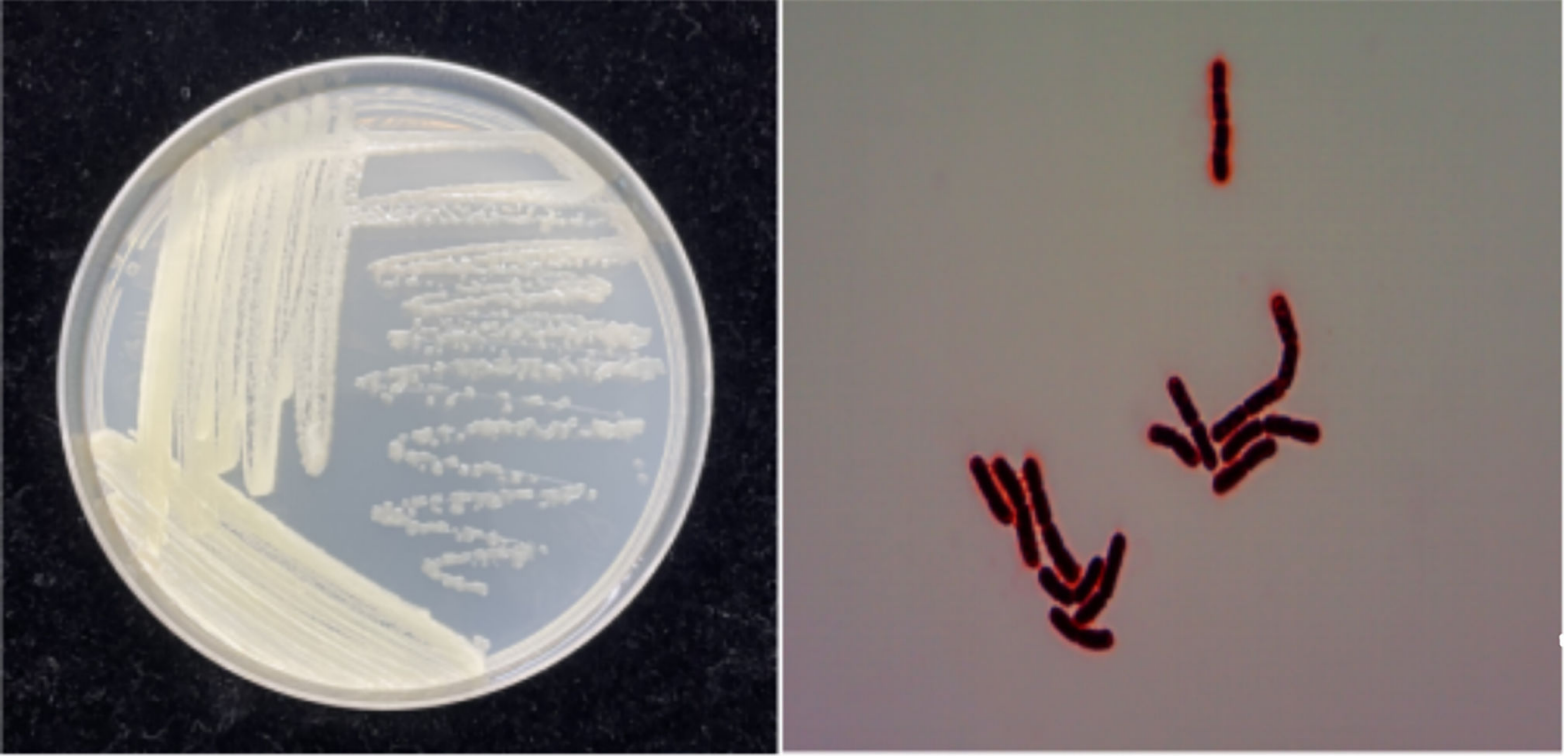
Colony morphology (left) and Gram staining (right) of the LL-8 strain.

**Fig 5:**
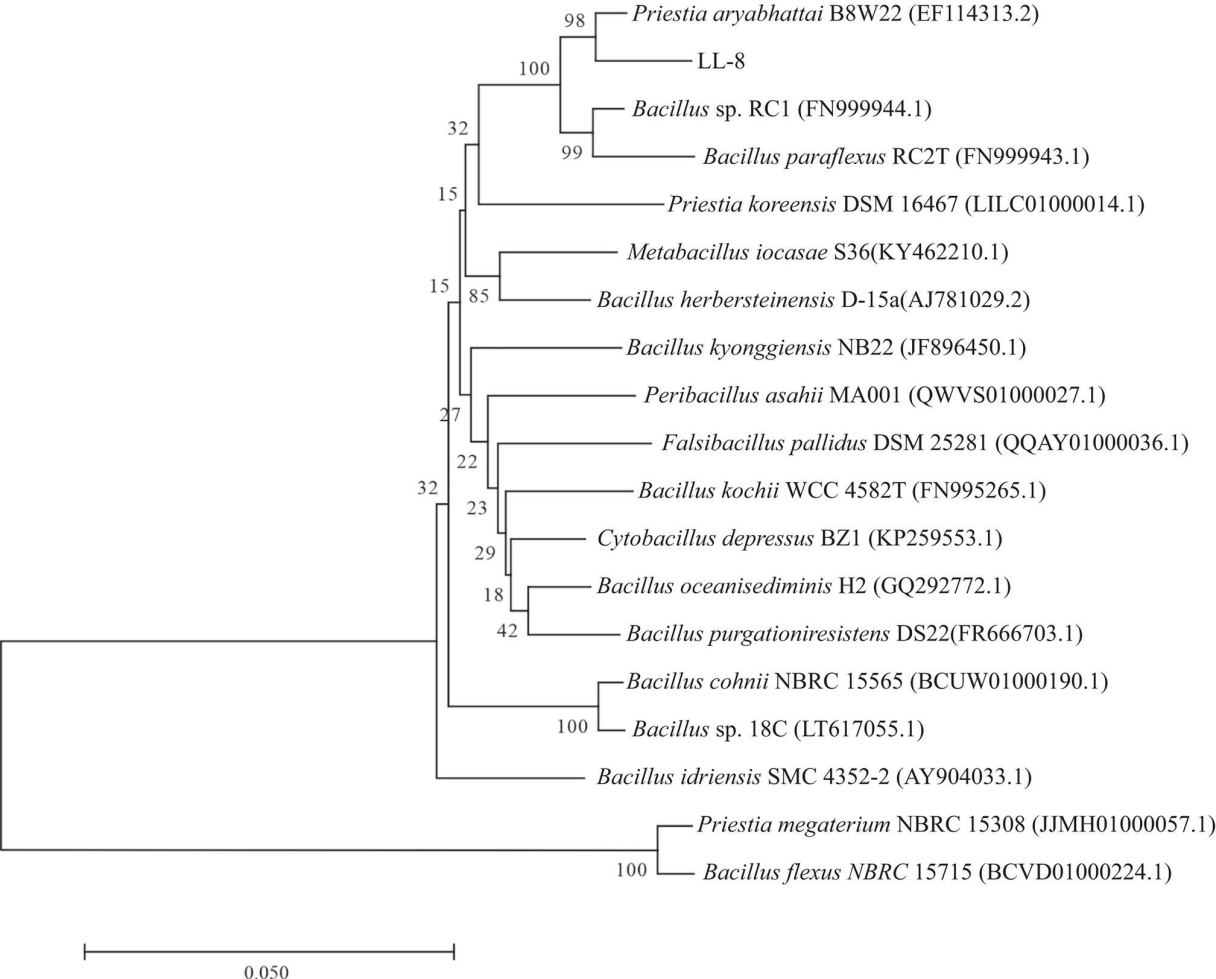
Phylogenetic tree of strain LL-8 based on the 16S rRNA gene.

LL-8 colonies were yellow with smooth and viscous surfaces and regular sides (Fig. 4). LL-8 cells were Gram-stain positive and rod-shaped. Phylogenetic analysis of the 16S rRNA gene sequence (comprising a 1,449 bp gene fragment) of strain LL-8 and known reference strains revealed that LL-8 exhibited the highest similarity with the strain type of *P. aryabhattai* B8W22^T^ (Fig. 5).

### Genomic analysis of strain LL-8

**Fig 6:**
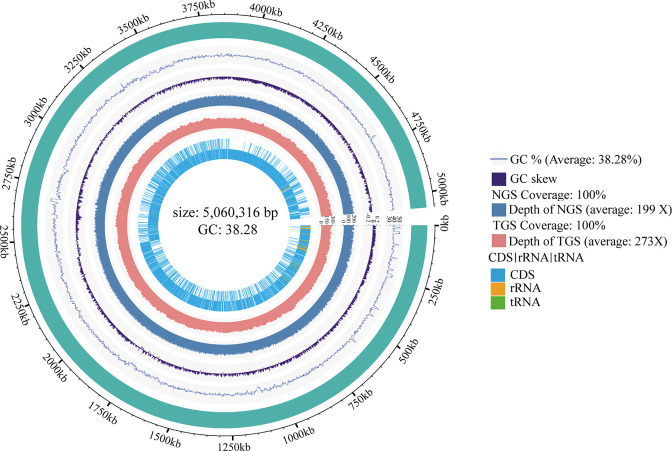
Genomic map of strain LL-8. From outside to inside, the first circle represents the genomic sequence information. The second circle represents the GC content of the genomic sequence with the dashed line indicating the average GC content of the reference genome. The third circle represents the GC skew curve of the genomic sequence with the dotted line showing a reference line with a GC skew of 0. The fourth circle represents the depth and coverage of the second-generation sequencing to display the reads coverage of different regions, whereas the dashed line shows the average reads coverage at the overall level. The fifth circle represents the depth and coverage information of the third-generation sequencing. The sixth circle shows the gene-coding regions (CDS) and non-coding RNA regions (rRNA, tRNA) in the reference genome, with the outer and inner layers representing the positive and negative strands, respectively.

The complete genome sequence of LL-8 was 5,060,316 bp in length, with a GC content of 38.28%. The genome encoded 5,346 genes with an average length of 893.52 bp, including 136 tRNA genes and 1 rRNA gene (Fig. 6). The whole genome shotgun project was deposited in the DDBJ/ENA/GenBank database under the accession JAZAQH000000000. The version described in this paper is JAZAQH010000000.

### GO annotation

**Fig 7:**
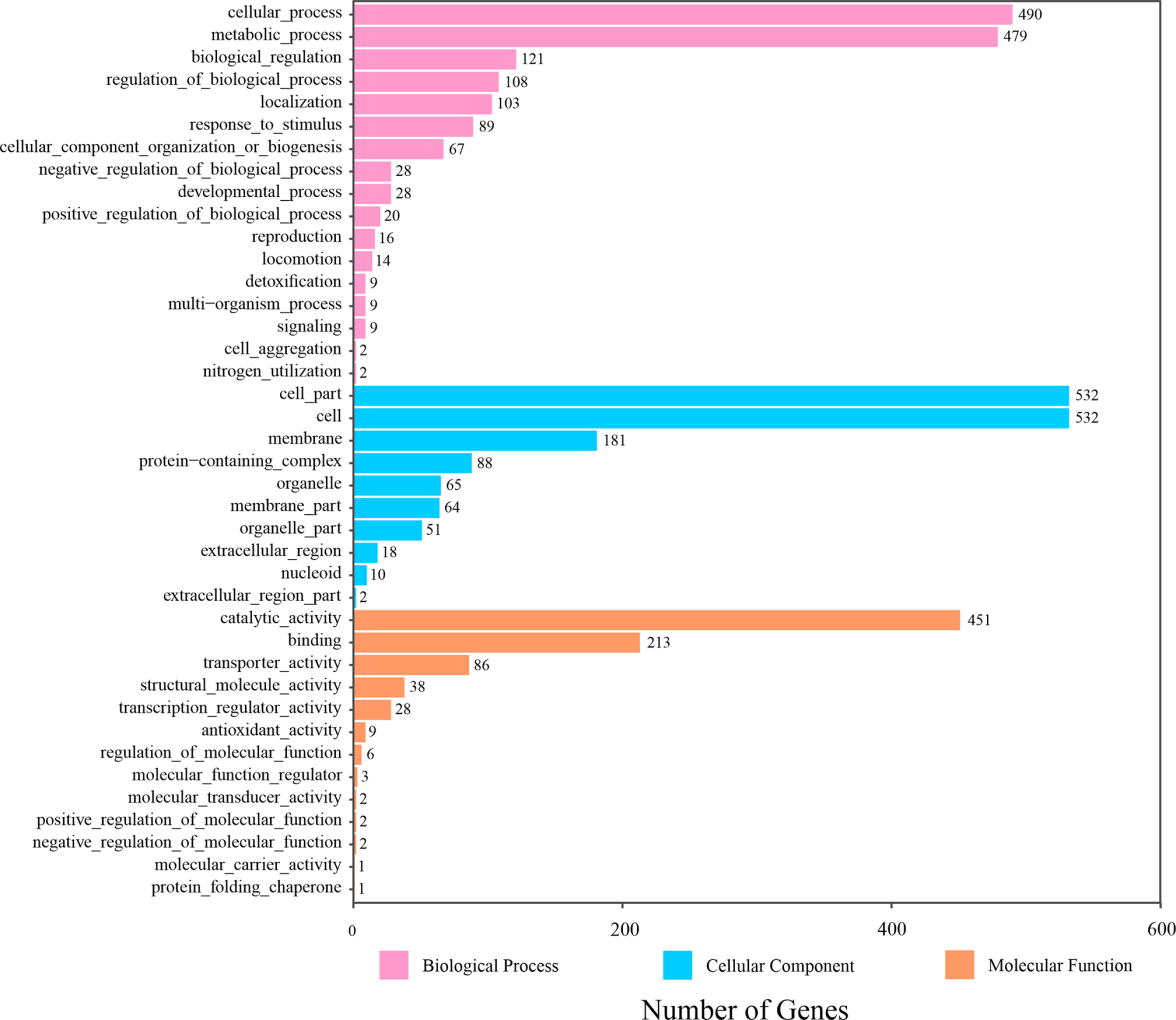
GO functional classification annotation of strain LL-8.

The distribution of functional genes encoded by strain LL-8 was identified by comparing its amino acid sequences against the GO database (Fig. 7). A total of 3,979 genes were annotated against the GO database across 40 total categories within the larger categories of biological processes, cellular components, and molecular functions that comprised 17, 10, and 13 sub-categories, respectively. In the biological process category, 1,594 genes were identified, with those in the cellular process and metabolic process categories being the most prevalent, comprising 490 and 479 genes, respectively. In the cellular component category, 1,543 genes were annotated, with the sub-categories of cell part, cell, and membrane comprising 532, 532, and 181 annotated genes, respectively. A total of 842 genes were annotated within the molecular function category, with most involved in the sub-categories of binding, catalytic activity, and transporter activity, comprising 213, 451, and 86 genes, respectively.

### COG functional annotations

**Fig 8:**
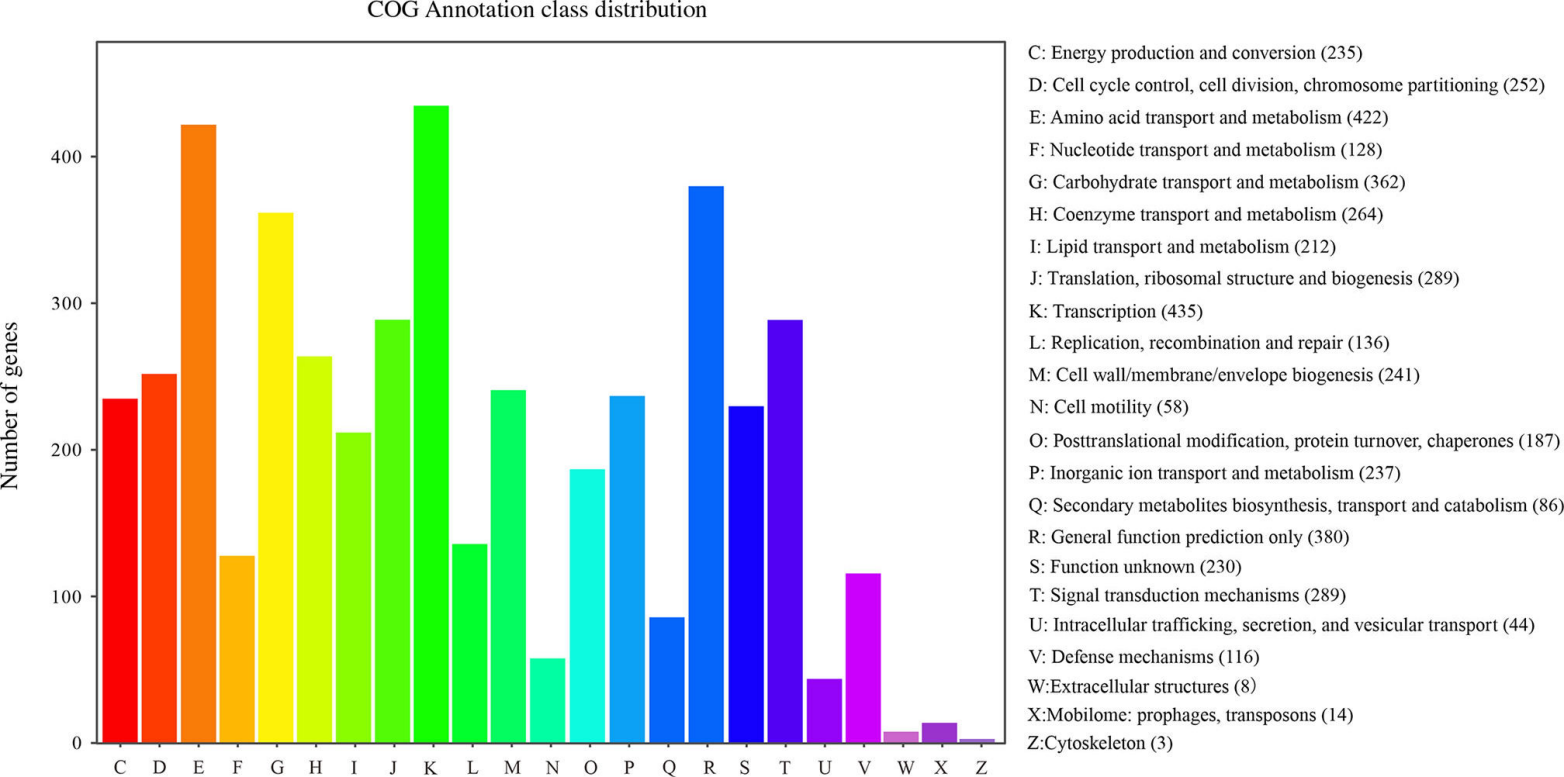
COG functional classification annotation of strain LL-8.

A total of 4,628 protein-coding genes were annotated and classified into 23 COG categories (Fig. 8). Transcription was the most prevalent annotation category, comprising 435 genes, accounting for 9.4% of all gene annotations. The category of amino acid transport and metabolism, in addition to the general function prediction-only category, comprised 422 and 380 genes, respectively, making up 9.1% and 8.2% of all annotations, respectively. Further, the category of carbohydrate transport and metabolism, in addition to the category of nucleotide transport and metabolism, comprised 362 and 128 genes, respectively, constituting 7.8% and 2.8% of all annotations, respectively. Thus, strain LL-8 exhibited promising potential for carbon and nitrogen metabolism. Other categories exhibited extensive annotations, including signal transduction mechanisms (289 genes, 6.2%), translation, ribosomal structure, and biogenesis (289 genes, 6.2%), and coenzyme transport and metabolism (264 genes, 5.7%).

### KEGG annotation and nitrogen metabolism functional genes

**Fig 9:**
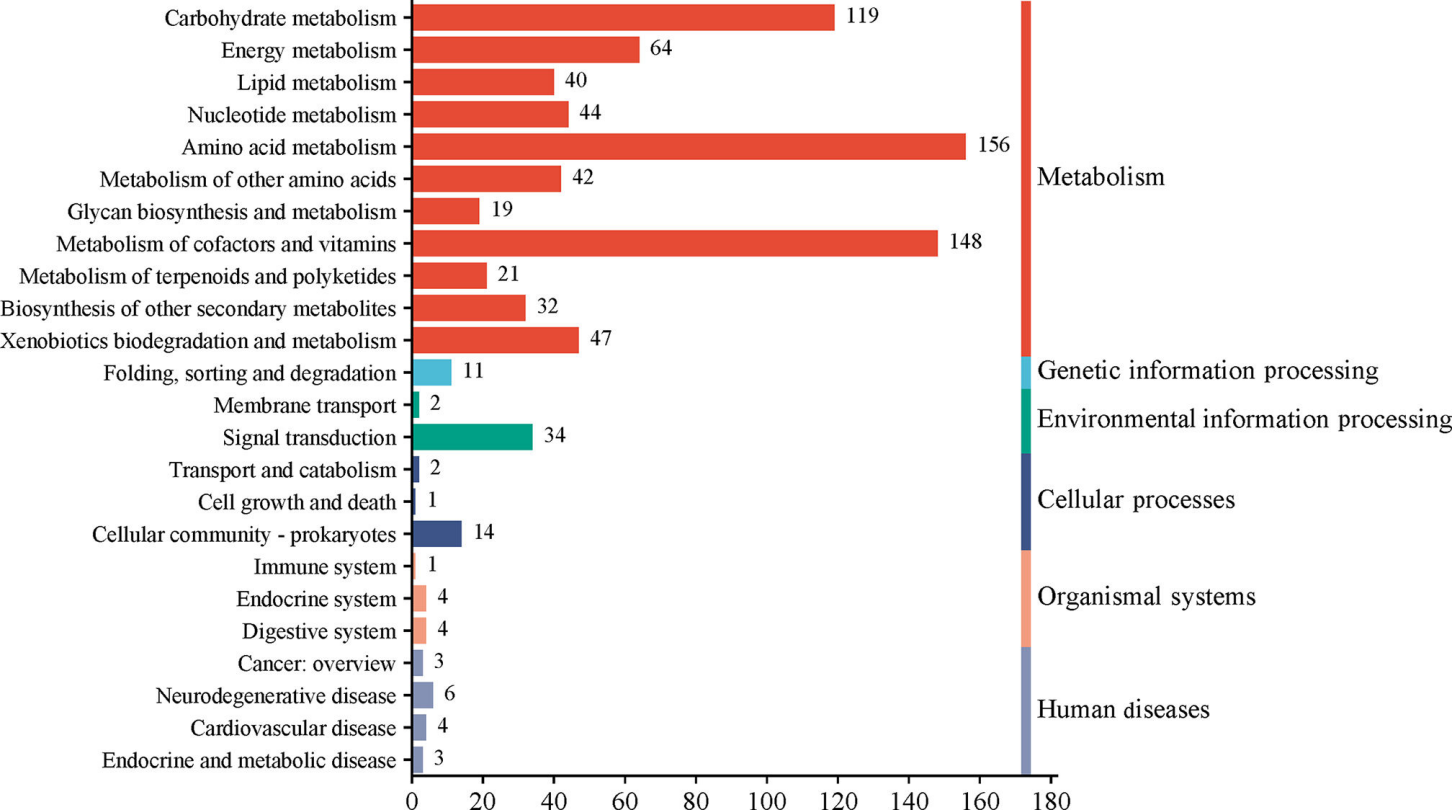
KEGG pathway classification of strain LL-8.

KEGG enrichment analysis (Fig. 9) revealed that 822 genes of LL-8 were mapped to six KEGG database metabolic pathways, comprising 27% of all annotated genes. Among these genes, 732 were annotated to the metabolism category. The two key metabolic pathway sub-categories were amino acid and carbohydrate metabolism, with 156 and 148 genes annotations, respectively, accounting for 18.98% and 18.00% of all KEGG annotations, respectively. Carbohydrate metabolism, energy metabolism, and xenobiotic biodegradation and metabolism pathways were also prevalent, comprising 14.48%, 7.8%, and 5.72% of the annotated genes, respectively.

**Fig 10:**
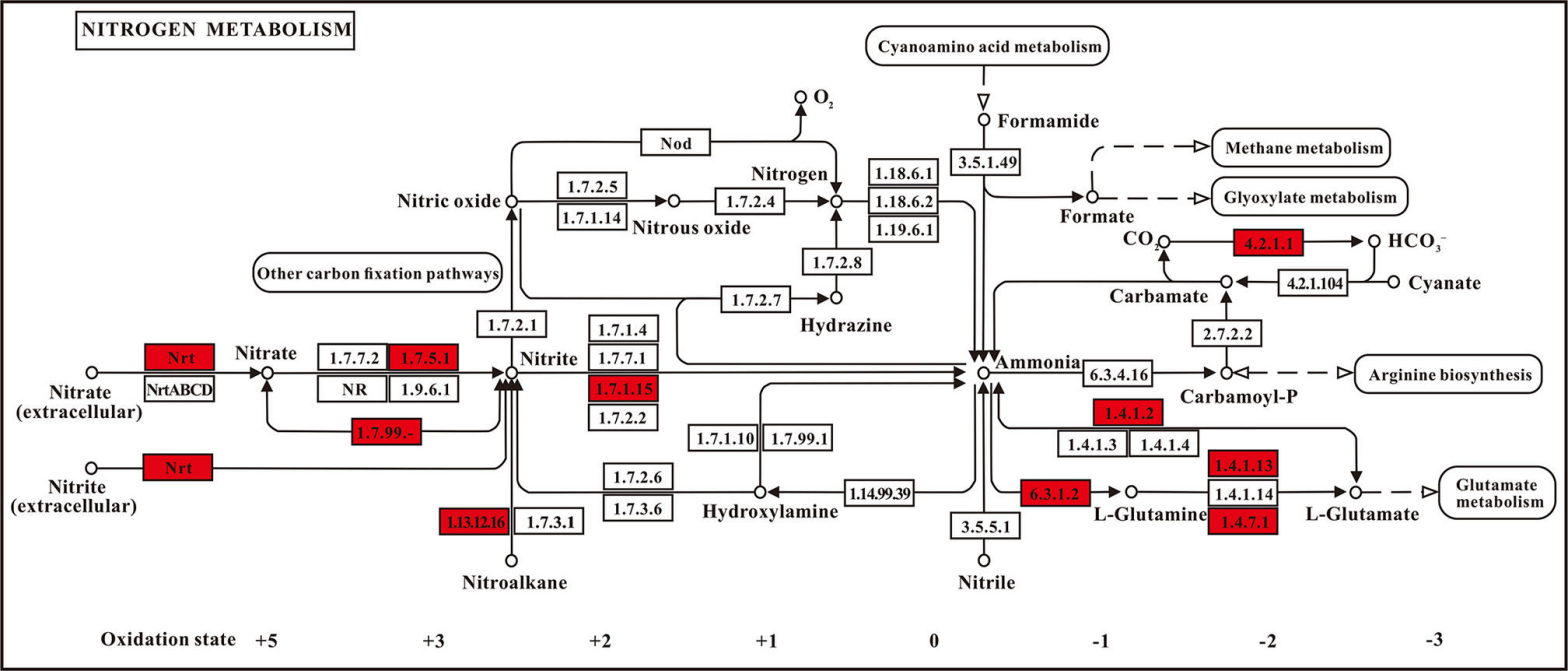
Partial nitrogen metabolism pathway of strain LL-8. Enzymes marked in red boxes are the annotated genes in the LL-8 genome with the EC (enzyme commission) number inside.

Further analysis revealed the presence of a few enzymes related to nitrogen metabolism (Fig. 10), including nitrate reductase (EC.1.7.5.1; EC.1.7.99.-), nitrite reductase (EC.1.7.1.15), glutamine synthetase (EC.6.3.1.2), glutamate synthase (EC.1.4.1.13), and glutamate dehydrogenase (EC.1.4.1.2). In addition, genes involved in nitrogen assimilation pathways were identified, including *gltB* (encoding glutamate synthase), *gltA* (encoding glutamate synthesize), *glnA* (encoding glutamine synthetase), and *gudB* (encoding glutamate dehydrogenase). Genes implicated in nitrate assimilation and reduction pathways, including n*asD*, *nasE*, *nasC*, *narK*, *nirB*, and *nirD*, were also identified across the LL-8 genome. Thus, strain LL-8 exhibited two primary nitrogen metabolic pathways, i.e., reduction of nitrate to ammonium and assimilation of ammonia.

## DISCUSSION

Total global solid waste stock will reach 3.4 billion tons by 2050 (21). Aerobic composting is an important way to reuse these organic wastes. However, nitrogen is readily lost during composting, leading to decreased compost quality and secondary environmental pollution, especially when compost temperatures are high. Several key processes related to nitrogen transformations occur during aerobic composting, including ammonification, nitrification, denitrification, and microbial assimilation (22). Among these processes, nitrification and microbial assimilation play critical roles in reducing NH_3_ volatilization during composting. It follows that identifying the ability of ammonium nitrification and assimilation of these bacteria in addition to analyzing their biological capacities could help reduce nitrogen volatilization loss during aerobic composting. Investigations of functional genes related to ammonium/ammonia conversion in composts have primarily focused on nitrification, denitrification, and nitrogen fixation. For example, biological nitrogen fixation is mediated by a nitrogen-fixing enzyme encoded by nifH, which can improve nitrogen quality in composts (23). Genes, such as *amoA*, *Hao*, *nirK*, *nirS*, and *nosZ*, have been widely studied as signature genes for nitrification and denitrification (9, 24, 25). However, it is difficult for most nitrifying bacteria to survive at the high temperature of composting, thereby limiting their efficient oxidation of NH_4_^+^-N to reduce NH_3_ volatile loss. In addition to reducing NH_3_ volatilization by oxidizing NH_4_^+^-N to NO3—N through the nitrification process, ammonia assimilation by bacteria can also reduce the loss of ammonia nitrogen by converting NH_4_^+^-N into organic nitrogen (20), which is also disclosed in this study.

Ammonia assimilation is the process by which microorganisms convert NH_4_^+^-N into glutamate through the two classical pathways encompassing glutamate dehydrogenase (GDH) and glutamine synthetase (GS)/glutamate synthetase (GOGAT) under the action of GS, GDH, and GOGAT (26, 27). In the GDH pathway, NH_4_^+^-N combination with α-ketoglutarate leads to glutamate production via catalysis by GDH. In the GS/GOGAT pathway, NH_4_^+^-N and glutamate are decomposed to glutamine due to GS hydrolyzation of ATP by GS, whereas GOGAT catalyzes the transformation of glutamine into glutamic acid and α-ketoglutaric acid and reduced NADH while concomitantly synthesizing nitrogen-containing organic compounds at the end of the process (28, 29).

Although there is limited research on the functional microorganisms and genes involved in ammonia assimilation during aerobic composting, several studies have revealed the important role of ammonia assimilation by functional bacteria in nitrogen retention and NH_3_ loss reduction. Zhu’s research revealed that ammonia assimilation contributed 53.4% for nitrogen preservation in composting by the glutamate dehydrogenase (GDH) pathway of 18 ammonia-assimilating bacteria, such as *Paenibacillus*, *Erysipelatoclostridium*, *Defluviimonas*, *Proteiniphilum*, *Brachybacterium*, *Lactobacillus*, and *Virgibacillus,* all of which could regulate nitrogen assimilation functional genes, such as *ans*B, *gud*B, *gln*A, and *glt*D (30). Numerous studies have shown that ammonium-assimilating microbial inoculants increased the NH_4_^+^-N absorption rate by 74% through regulating the expression of *gdhA* and *glnA* (31, 32). Thus, nitrogen can be effectively retained by applying exogenous ammonia-assimilating functional bacteria or by modulating the regulation of ammonia-assimilating functional genes, such as *glnA* and *gltB*.

Here, a thermophilic ammonia-converting bacterium (strain LL-8) was screened and identified. It could convert ammonia up to 32.7% in ammonia-oxidizer culture medium at 50°C (Fig. 2) and reduce 42.9% of NH_3_ loss in chicken manure composting from the high-temperature stage (Fig. 3). It was found to be highly similar to *P. aryabhattai* B8W22^T^ by phylogenetic tree analysis, consequently being identified as *P. aryabhatta* (Fig. 5), which was named *Bacillus aryabhattai* before (33). Strains in this species have been used in repairing phenol pollution of wastewater (34), producing indole-3-acetic acid (IAA) in rice (35), solubilizing phosphate and fixing nitrogen in maize (36), as well as manipulating the glyphosate herbicide residues in environments for bioremediation (37). All of the above implied that this species is safe and harmless for agriculture and environment. Previous reports have also highlighted the role of *P. aryabhattai* in nitrogen transformations. For example, strain B8W22 was able to degrade NO2—N (38). Further*, P. aryabhattai* KX-3 converted NH_4_^+^-N into NO3—N or NO2—N via heterotrophic nitrification at a high conversion rate of 92.5%. Kang et al. (39) identified the nitrogen degradation pathway of *P. aryabhattai* KX-3 by analyzing the functional enzymes (nitrite reductase [NIR], nitratase [NR], and ammonia monooxygenase [HAO]) and corresponding genes (*amoA*, *hao*, *napA*, *nirS*, and *nosZ*). Moreover, nirB/D, glnA, and gltB/D were shown to be upregulated in the presence of abundant NH_4_^+^-N, confirming that the strain removed NH_4_^+^-N through assimilation and heterotrophic nitrification. In this study, several genes related to nitrogen metabolism were identified in the genome of LL-8, including the glutamine synthetase-encoding gene *glnA* (40), glutamate synthase-encoding genes *gltA* and *gltB* (41), glutamate dehydrogenase-encoding genes *gudB* and *rocG* (42), nitrate transport-associated gene *narK* (43), nitrite reductase-encoding genes *nasD* and *nasE* within the same gene cluster, and assimilatory nitrate reductase-encoding gene *nasC* (44). However, genes that could encode enzymes, such as AMO, HAO, Nap, Nos, and Nor, involved in various nitrification and denitrification processes were not identified in LL-8 genome.

**Fig 11:**
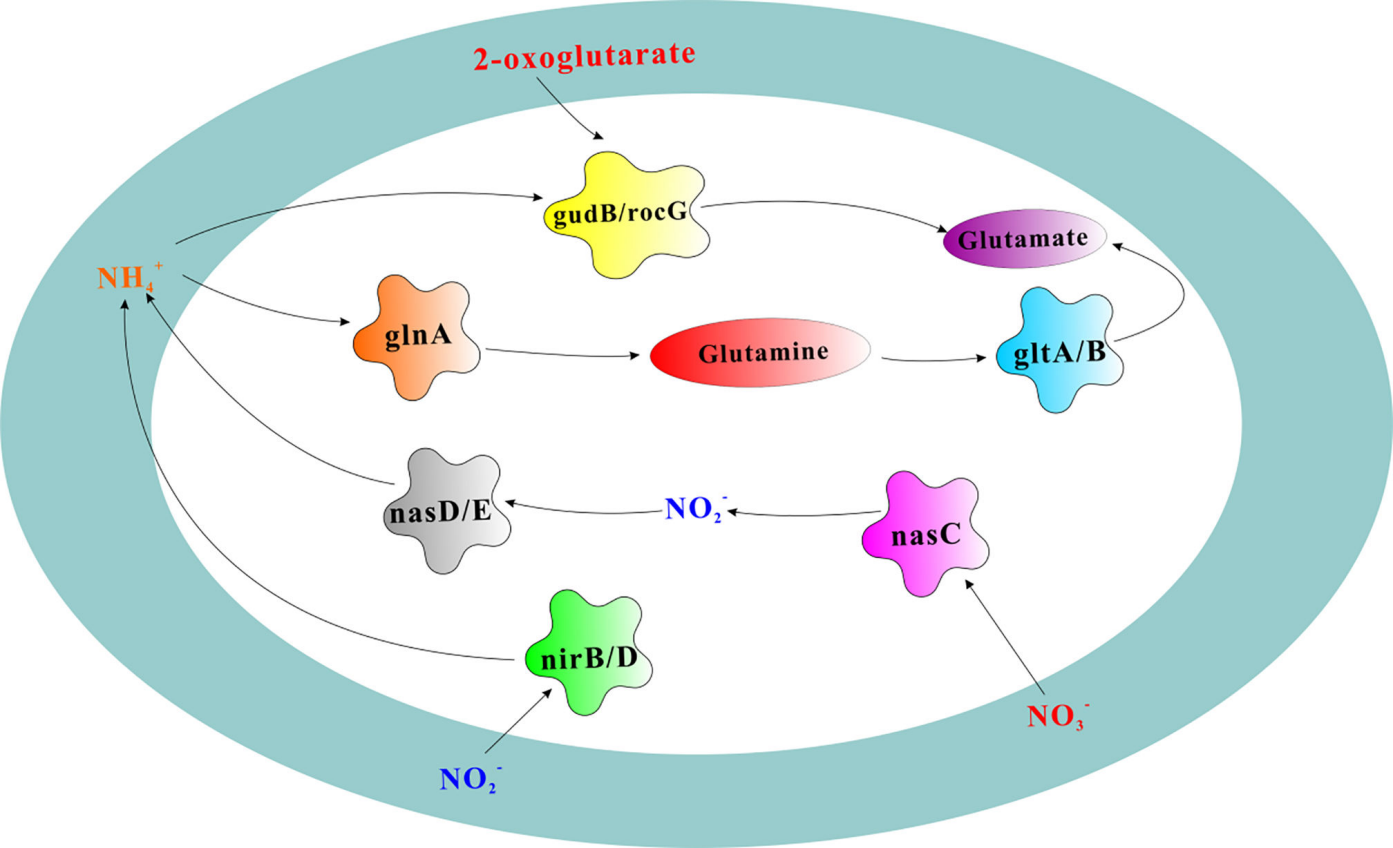
Nitrogen metabolism pathway of strain LL-8. The pentagram represents the functional gene, and the ellipse represents the product.

The changes of inorganic N forms in the test of NH_4_^+^-N conversion ability of the preliminary screened strains at high temperature seemed to be a coincidence with this view. The content of NO3—N in each inoculating treatment was always lower than that in CK, which only had 0.5-mL sterilized LB culture medium, whereas the content of NO2—N was in reverse when compared with CK (Fig. 2). It indicated that some NO3—N originating from LB was possibly transformed to NO2—N by these testing strains. Because LL-8 has no denitrification gene, it is speculated that this transformation in LL-8 treatment might result from the assimilation nitrate reductase encoded by *nasC*. At the same time, the cumulative reduction of NH_4_^+^-N, which is the nitrogen source of the ammonia-oxidizing bacteria medium in each inoculating treatment, gradually increased with the culturing days, and that of LL-8 treatment was two times greater than the sum increment of NO3—N and NO2—N, but the total nitrogen content was stable in the entire culture. It could be speculated that there was another way to convert ammonia in LL-8 treatment. Because LL-8 had several genes related to ammonia assimilation as we discussed above, nitrogen conversion by LL-8 strain might be started by glutamine synthetase that was encoded by *glnA* gene to convert ammonium to L-Glutamine, then L-glutamine was catalyzed to L-glutamic acid by glutamate synthetase encoded by *gltA/gltB*; on the other hand, glutamate dehydrogenases encoded by *gudB/rocG* could catalyze ammonia and α-ketoglutarate to form glutamic acid (Fig. 11). The glutamic acid synthesized by both above pathways would form microbial available nitrogen and cellular compounds furthermore to maintain normal metabolism and complete life cycle (45, 46). All of the above implied that ammonia assimilation was most likely the main nitrogen conversion process of LL-8 treatment, and *P. aryabhattai* LL-8 is an ammonia-assimilation bacterial strain instead of ammonia-oxidizing bacteria.

In the early stage of aerobic composting, due to the mineralization of organic nitrogen, the content of NH_4_^+^-N increased rapidly (47). Coupled with the increase of pH and temperature, the NH_4_^+^-N was quickly converted into NH_3_ and loss in air in the early stage of high temperature. Chen et al. (48) and Meng et al. (49) revealed that the genes, such as *glnA* and *gltA/gltB*, in composting could be increased by increasing ammonia assimilation bacteria, which could promote the activity of GS and GOGAT enzymes to assimilate ammonia/ammonium and result in less volatile loss of ammonia. This might be the reason why LL-8 could significantly reduce NH_3_ volatilization in aerobic composting at high temperature.

In conclusion, strain LL-8 is a thermophilic bacterium of *P. aryabhattai* that can survive in high temperatures above 50°C. Genes involved in ammonia assimilation pathways (*gltB*, *gltA*, *glnA*, and *gudB*) and in nitrate assimilation and dissimilation reduction pathways (*nasD*, *nasE*, *nasC*, *narK*, *nirB*, and *nirD*) were annotated in LL-8 genome, whereas no genes involved in the nitrification and denitrification pathways were found. It is an ammonia-assimilating bacteria that significantly converted ammonium in culture medium with a rate of 32.7% at high temperature (50°C) and reduced NH_3_ volatilization with a rate of 42.9% in chicken manure aerobic composting when it was inoculated at the high-temperature stage. These results provide a theoretical foundation for the application of strain LL-8 in high-temperature composting, and it is a safe and promising microbial nitrogen retention additive to reduce nitrogen volatilization loss and environmental pollution.

## MATREIALS AND METHODS

### Screening of thermophilic ammonia-converting bacteria

Fourteen strains named as L1, L2, L5, L6, L7, LL-8, L13, L17, L19, L20, L21, L22, L23, and L25 were isolated from the high-temperature stage (>50°C) mixture of livestock manure aerobic composting using a specific ammonia-oxidizing bacteria medium ((NH_4_)_2_SO_4_ 0.50 g/L, NaCl 2 0.00 g/L, FeSO_4_·7H_2_O 0.40 g/L, K_2_HPO_4_ 1.00 g/L, CaCO_3_ 5.00 g/L, MgSO_4_ 0.50 g/L, pH 7.20, autoclaved at 121°C for 30 min). After activation in LB solid medium (Beef extract 3.0 g/L, Peptone 10.0 g/L, NaCl 5.0 g/L, agar 20 g/L, pH 7.20, autoclaved at 121°C for 30 min), one colony for each strain was inoculated into 25 mL of ammonia-oxidizing bacteria medium and incubated at 150 rpm and 50°C. The experiment was arranged completely randomly with three replicates. After 15 days of cultivation, culture suspensions were sampled to preliminarily screen the thermophilic ammonia-converting bacteria by measuring NH_4_^+^-N content and analyze the converting rate of NH_4_^+^-N using the following formula:


$$A\left(mg/L/d\right)=\frac{N_{0}-N}{T}$$

where A is the NH_4_^+^-N converting rate, N_0_ is the NH_4_^+^-N concentration of the control treatment CK (mg/L), N is the NH_4_^+^-N concentration of the inoculated group (mg/L), and T is the cultivation time (15 days).

### Ammonium conversion ability of thermophilic ammonia-converting bacteria

The preliminary screened strains (LL-8, L17) were inoculated in LB liquid medium (Beef extract 3.0 g/L, Peptone 10.0 g/L, NaCl 5.0 g/L, pH 7.20, autoclaved at 121°C for 30 min) and incubated at 50°C and 150 rpm for 12 –16 h to obtain a strain agent with 10^8^ CFU/mL. Then, the agent was inoculated into ammonia-oxidizing culture medium to incubate 7 days at 50°C and 150 rpm. Four kinds of inocula became the treatments: CK, inoculated 1% sterilized LB liquid medium; LL-8, inoculated 1% LL-8 strain agent; L17, inoculated 1% L17 strain agent; LL-8+L17, inoculated 1% LL-8 and 1% L17 strain agent. Triplicate suspension samples were randomly collected every 24 h in the incubating process. The contents of NH_4_^+^-N, nitrate nitrogen (NO3—N), and nitrite nitrogen (NO2—N) were investigated to analyze NH_4_^+^-N conversion abilities of the preliminary screened strains and to identify the candidate thermophilic ammonia-converting bacteria with the strongest ability. The cumulative reduction of NH_4_^+^-N (mg/L) represents the difference of NH_4_^+^-N concentration (mg/L) between the first day and the test day.

Initially, the nitrogen contents of LB liquid medium were as follows: total nitrogen (TN) 3.5 g/L, NH_4_^+^-N 13.8 mg/L, NO3—N 2.18 g/L, and NO2—N 1.22 g/L. The NH_4_^+^-N content of ammonia-oxidizing culture medium was 136.8 mg/L.

### Composting with the candidate bacteria

An aerobic composting was conducted with the device for solid waste high-temperature aerobic composting (patent number: ZL 201010589910X) to test the effects of the candidate thermophilic ammonia-converting bacteria (LL-8) on reducing ammonia (NH_3_) volatilization loss in composting. Chicken manure (total organic carbon [C] 206.70 g /kg, total nitrogen [N] 15.57 g /kg, C/N ratio 13.2, and moisture content 42.20%) and herbal medicine residue (C 296.32 g /kg, N 10.22 g /kg, C/N ratio 29, and moisture content 26.65%) were used as the raw materials.

Two treatments were set up: LL-8 treatment was applied with 2% (v/w) LL-8 strain agent, and CK treatment was applied with 2% (v/w) sterile LB liquid medium at the same time. Each treatment was repeated thrice with a completely random arrangement. When composting, the C/N ratio of chicken manure was adjusted to 25:1 by herbal medicine residue, and 10-kg mixture was loaded into each device. Ventilation was 30 min/2 h, 4 L/min. Volatile NH_3_ was absorbed by 20 g/L boric acid, which was changed by a new one once a day before the high-temperature stage and then once every 2 days. The mixture and ambient temperature were monitored with the ZDR-40 data recorder (Hangzhou Zeda Instruments Co., Ltd., China). The NH_3_ volatilization was measured until the temperature of composting mixture dropped to the ambient temperature and no NH_3_ volatilization was detected.

### DNA extraction and genome sequencing

The candidate strain was inoculated into LB medium and incubated at 37°C and 180 rpm for 12 h. The culture suspension was centrifuged at 10,000×*g* for 3 min to collect cells that were immediately frozen in liquid nitrogen. DNA was extracted using SDS extraction method combined with column purification, then checked with 1% agarose gel electrophoresis (200 V, 30 min), and quantified using Qubit. DNA sequencing was performed at Suzhou Panomic Biomedical Technology Co., Ltd. by using an R9.4 sequencing chip and a PromethION sequencer (Oxford Nanopore Technologies, Oxford, UK).

### Evolutionary analysis

16S rRNA gene sequences were compared against the NCBI 16S rRNA gene database (http://www.ezbiocloud.net/eztaxon/) using an identification threshold >96% nucleotide identity. Eighteen most similar 16S rRNA gene sequences were selected for phylogenetic tree construction using Maximum Likelihood methods after multiple sequence alignment using the Mega seven software program.

### Gene annotation

GO (50), COG (51), and KEGG (52) annotations were evaluated to identify the functions of protein-coding genes using the NCBI Blast+, Uniprot, and KEGG Automatic Annotation Server (KAAS) annotation platforms.

### Statistical analyses for culturing and composting work

Statistical difference (*P* < 0.05) among treatments were performed in DPS v19.5 (53) with one-way analyses of variance (ANOVA, Duncan’s method) for culturing work and with two-samples *t*-test for composting work.
